# Influence of Thread Pitch, Helix Angle, and Compactness on Micromotion of Immediately Loaded Implants in Three Types of Bone Quality: A Three-Dimensional Finite Element Analysis

**DOI:** 10.1155/2014/983103

**Published:** 2014-07-08

**Authors:** Pan Ma, Wei Xiong, Baosheng Tan, Wei Geng, Jiaqiang Liu, Weihong Li, Dehua Li

**Affiliations:** ^1^Department of Oral Implantology, Beijing Stomatological Hospital of Capital Medical University, Beijing 100050, China; ^2^Department of Oral Implantology, School of Stomatology, State Key Laboratory of Military Stomatology, The Fourth Military Medical University, Xi'an, Shanxi 710032, China; ^3^Clinical Aviation Medicine Center of PLA, Air Force General Hospital, Beijing 100142, China; ^4^Department of Oral and Cranio-Maxillofacial, Ninth People's Hospital, Shanghai Jiao Tong University School of Medicine, Shanghai 200011, China; ^5^School of Basic Medical Science, Capital Medical University, Beijing 100069, China

## Abstract

This study investigated the influence of thread pitch, helix angle, and compactness on micromotion in immediately loaded implants in bone of varying density (D2, D3, and D4). Five models of the three-dimensional finite element (0.8 mm pitch, 1.6 mm pitch, 2.4 mm pitch, double-threaded, and triple-threaded implants) in three types of bone were created using Pro/E, Hypermesh, and ABAQUS software. The study had three groups: Group 1, different pitches (Pitch Group); Group 2, same compactness but different helix angles (Angle Group); and Group 3, same helix angle but different compactness (Compact Group). Implant micromotion was assessed as the comprehensive relative displacement. We found that vertical relative displacement was affected by thread pitch, helix angle, and compactness. Under vertical loading, displacement was positively correlated with thread pitch and helix angle but negatively with compactness. Under horizontal loading in D2, the influence of pitch, helix angle, and compactness on implant stability was limited; however, in D3 and D4, the influence of pitch, helix angle, and compactness on implant stability is increased. The additional evidence was provided that trabecular bone density has less effect on implant micromotion than cortical bone thickness. Bone type amplifies the influence of thread pattern on displacement.

## 1. Introduction

In the conventional protocol for implant-based dental repair, an undisturbed 3- to 6-month healing period is suggested for successful implant osseointegration [1–3]. Although this approach has been shown to be highly predictable and successful, the extended treatment period may be perceived as a considerable inconvenience. Because many patients expect immediate rehabilitation [4], considerable effort has been directed toward investigating the effect of immediate loading to dental implants.

Restoration that allows immediate loading of implants in edentulous areas is increasing because of the advantages of regained chewing functions and aesthetics. Although studies have found that the survival rate of immediately loaded implants is acceptable [5, 6], for single-tooth restorations, an immediately loaded implant is still considered to have a higher risk of failure and a lower success rate [7, 8]. These drawbacks might be due to increased micromovement at the bone-implant interface (BII), which leads to fibrous encapsulation around the implant rather than full osseointegration [9]. Any motion greater than 100 *μ*m during the healing process may affect the osseointegration of implants [10]. Pilliar et al. and Viceconti et al. [11, 12] identified that micromotion of more than 150 to 200 *μ*m will lead to failed osseointegration, whereas elsewhere [13], a maximum limit of 150 *μ*m has been reported for micromotion to ensure the success of implants.

Consequently, modifications in implant body design have been suggested to increase the success of immediate loading by gaining better initial stability and restricting micromovement. Threads are used to maximize initial contact, improve initial stability, enlarge implant surface area, and favor the dissipation of interfacial stress [14, 15]. In addition, thread depth, thickness, face angle, pitch, and helix angle are some of the geometric patterns that can be varied to alter the functional thread surface and affect the biomechanical load distribution of the implant [16, 17]. The need for a better design of thread is thus required for commercial implant systems.

Among the implant design variables, the pitch is considered to have a significant effect on stability, because of its effect on surface area [18]. Akkocaoglu and colleagues found that clinical advantage of Straumann's synOcta ITI TE implant is the decreased thread pitch distance (0.8 mm) and the increased compactness of the thread, which can decrease the degree of micromovement in the bone [19]. Implants with different pitches have two major differences: one is the helix angle, which increases with an increase in pitch, whereas the other is the compactness, which becomes more sparse as the pitch increases. However, there is no strong scientific evidence noted in the literature to elucidate the effects of helix angle and thread compactness on the primary stability of immediately loaded dental implants in bone of distinctly different density.

Primary stability of immediately loaded dental implants is related to micromotion. Clinically, it is impossible to introduce a device into an implant-bone interface to investigate the level of micromotion between the bone and the implant under masticatory force. Several parameters have been used in past experimental and clinical studies to represent implant primary stability in experimental or clinical studies. These parameters include insertion torque [20], removal torque [21], cutting torque [22], pull-out force [21], Periotest data [21, 23], and implant stability quotient (ISQ), as derived from resonance frequency analysis [24, 25]; however, it would appear that most of these parameters are either too low in sensitivity or that the correlation between these parameters is somewhat questionable [22, 26]. Under such circumstances, finite element analysis (FEA) is an efficient technique for the evaluation of micromotion [27–29]. The purpose of this study was to compare the biomechanical effects of implant thread pattern (thread pitch, helix angle, and compactness) on micromotion in three types of bone (D2, D3, and D4) by means of three-dimensional (3D) FEA and to evaluate the complex, irregular structures by nonlinear FEA.

## 2. Materials and Methods

In this study, the effects of single-threaded, double-threaded, and triple-threaded implants on micromotion at the bone-implant interface were examined using the 3D FEA method.

### 2.1. 3D Model of the Bone-Implant System

**Figure 1:**
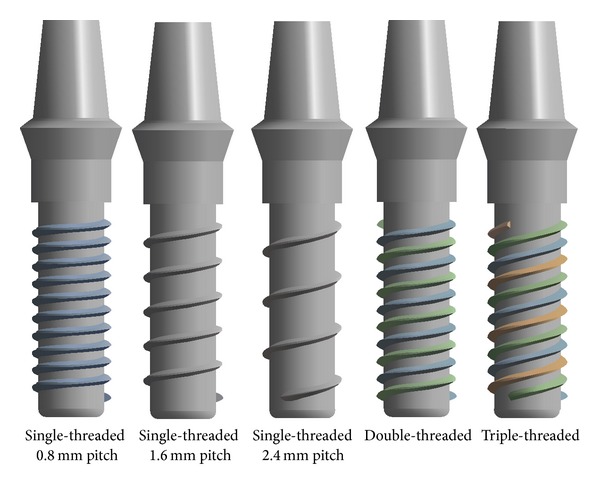
Five different configurations of implants with abutment.

The 3D models of a mandibular bone block and a screw-shaped dental implant with a healing abutment were constructed using computer assisted designing (CAD) system (PRO/E) on a personal computer. To study the thread helix angel and the compactness of the single-threaded, double-threaded, and triple-threaded implants, five different V-shaped threaded implants were designed: single-threaded implants with pitches of 0.8 mm, 1.6 mm, and 2.4 mm; a double-threaded implant with a thread pitch of 1.6 mm and thread spacing of 0.8 mm; and a triple-threaded implant with a thread pitch of 2.4 mm and thread spacing of 0.8 mm (Figure 1). For analysis, we compared these designs as three groups: Group 1, or the Pitch Group, had different pitches, including single-threaded implants with pitches of 0.8 mm, 1.6 mm, and 2.4 mm; Group 2, or the Angle Group, had the same thread compactness but different helix angles, including double-threaded, triple-threaded implants, and single-threaded implant with 0.8 mm pitch; and Group 3, or the Compactness Group, had the same helix angle and different thread compactness, including double-threaded and single-threaded implants with 1.6 mm pitch and triple-threaded and single-threaded implants with 2.4 mm pitch (please refer to Table 1). All implants were designed with a 3.7 mm tip diameter, 10.0 mm threaded length, and 0.3 mm collar height (helix angle, *α* = *β* = 60°).

**Figure 2:**
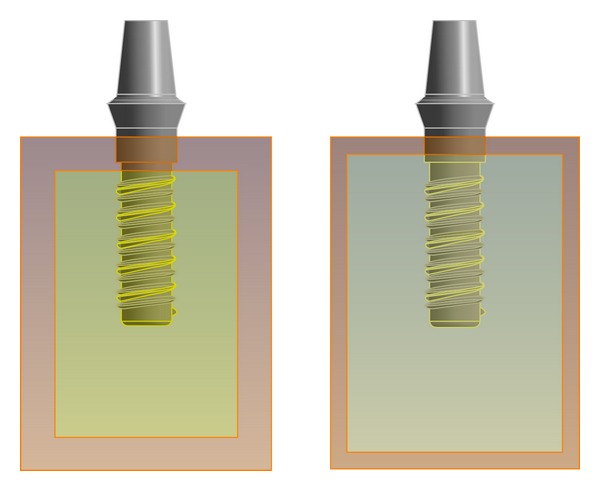
Configuration of the dental implant/bone system. In the D2 model, a core of dense cancellous bone was covered by a thick layer of compact bone with a width of 2 mm. The geometric configurations of D3 and D4 models were similar to those of the D2 model, but the width of the compact bone layers was reduced to 1 mm.

The five 3D solid V-threaded implants were modeled under similar conditions. The bone block had dimensions of 15 mm × 25 mm × 20 mm representing buccolingual × mesiodistal × inferosuperior surfaces. As per the classification of Lekholm and Zarb [33], there are four distinctly different bone qualities (D1, D2, D3, and D4). In this study, we simulated the latter three types (D2, D3, and D4). D1 bone quality was not simulated as it consists of compact bone only. In the D2 model, a core of dense cancellous bone was covered by a thick layer of compact bone with a width of 2 mm. The geometric configurations of D3 and D4 models were similar to those of the D2 model, but the width of the compact bone layers was reduced to 1 mm. In the D3 model, a thin layer (1 mm) of cortical bone surrounds a core of dense trabecular bone of favorable strength (Figure 2). In the D4 model, a thin layer (1 mm) of cortical bone surrounds a core of low-density trabecular bone. The thickness of the compact bone set in the 3D models was based on previous studies [34–36]. In the above models, the mesial and distal sides were not covered by compact bone.

**Figure 3:**
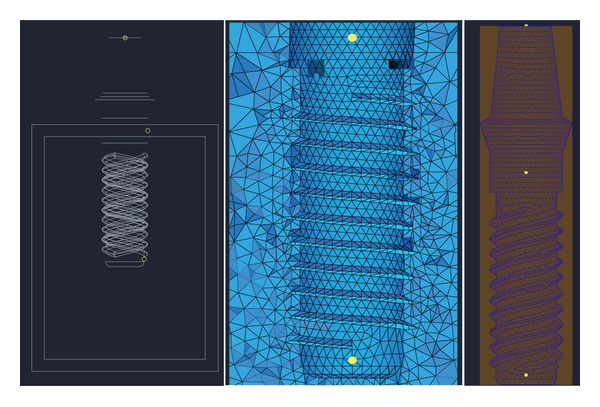
Four coordinate points were defined to the collar, tip, and corresponding bone quality of each type of implant model. Four coordinate points were nodes in model meshing, with each point located in the same position in each model.

The combined solid model was transferred to Hypermesh 7.0 (Hypermesh 7.0 Inc., Providence, RI, USA) to create a finite element meshed model for later analysis. To guarantee the comparability of the model, four coordinate points were defined to the collar, tip, and corresponding bone type (compact and cancellous) in each style of implant model, and the points in all of the implant models were the same for the collar and the tip, respectively. The four coordinate points are nodes in the model meshing, with each point located in the same position in each model (Figure 3). The tetra meshing was chosen to refine the implants and bone interfaces (Figure 3). Each mathematical model included approximately 147256–163298 nodes and 83710–92522 solid elements. These models were then input into the finite element package (ABAQUS Inc., Providence, RI, USA). The accuracy of a 3D finite element model is related to the element mesh density in relation to the element configuration chosen. This can be assessed objectively by repeated calculations for increased mesh refinement and checking the convergence of the micromotion results. The convergence criteria were set as the change of displacement variations of <3% for models with different element sizes.

### 2.2. Material Properties

The mechanical properties of the models were assumed to be homogeneous, isotropic, and linearly elastic. The specific values of the properties were adopted from previous studies [30–32] and are listed in Table 2.

### 2.3. Interface Conditions

Nonlinear frictional contact elements were used to simulate the adaptation between the bone and the implant. A frictional coefficient of 0.3 was assumed for all contact surfaces [37–39]. The amount of interfacial sliding between the contact elements was calculated and analyzed.

### 2.4. Constraints and Loads

The boundary condition of total fixation on the nodes of the three faces (the mesial, distal, and bottom faces) of the bone block was chosen [38]. Forces of 200 N were applied along the axis of the implant and forces of 100 N at an angle of 90 degrees in the buccolingual direction to the center of the abutment superstructure. The resultant axial load (200 N) corresponded approximately to the average maximum occlusal force that has been reported by Mericske-Stern and Zarb [40] for fixed partial prosthesis supported by implants in the molar region. Graf et al. [41] measured lateral forces in the molar region that were up to half of the axial loads; thus, we chose a buccolingual component of 100 N for this study.

### 2.5. 3D Finite Element Evaluations on Implant Micromotion

*Definition of Implant Micromotion*. By the influence of the loaded force, the relative displacement between the nodes of the implant surface and the corresponding bone interface is represented as the relative displacement at a 3D coordinate, including the* X* axis (buccolingual direction), the* Y* axis (vertical direction), and the* Z* axis (mesiodistal direction) and the comprehensive relative displacement, which is the square root of the sum of the square of implant motion at three-dimensional directions. If the motion of implant surface node is marked as *dX*_1_, *dY*_1_, and *dZ*_1_ at the coordinate and the motion of corresponding bone interface node is marked as *dX*_2_, *dY*_2_, and *dZ*_2_, the comprehensive relative displacement is

(1) $$\begin{array}{c} \left(S\right)=\sqrt{{\left(dX_{1}-dX_{2}\right)}^{2}+{\left(dY_{1}-dY_{2}\right)}^{2}+{\left(dZ_{1}-dZ_{2}\right)}^{2}}. \end{array}$$

## 3. Results

### 3.1. Convergence Test

**Figure 4:**
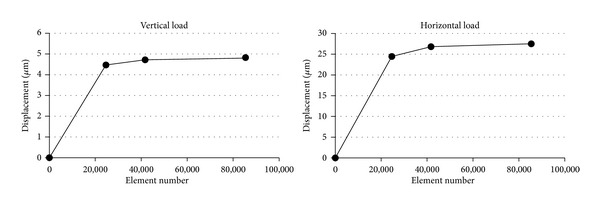
Convergence test results in Single 0.8 mm pitch model in D2 bone.

Convergence testing of the 3D finite element models was performed to verify the accuracy of the mesh; this resulted in a convergence criterion of less than 3% change in the maximum displacement of bone between the elements at a given point (Figure 4).

### 3.2. Vertical Load

**Figure 5:**
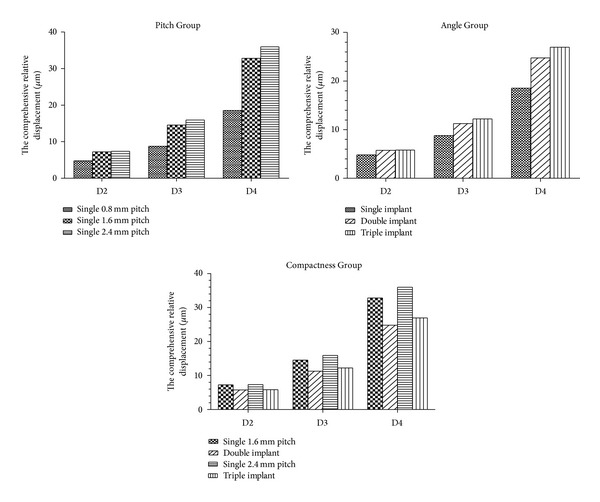
Comprehensive relative displacement among the Pitch Group, the Angle Group, and the Compactness Group under vertical load.

For the displacement of the five implants under vertical load, please refer to Table 3 and Figure 5. The results of each group are described separately.

(1) In the Pitch Group, the 0.8 mm pitch single-threaded implant had a minimum comprehensive relative displacement in the collar region of the implant, whereas the 2.4 mm pitch single-threaded implant had the maximum. Compared with the 0.8 mm pitch single-threaded implant, the comprehensive relative displacement of the 1.6 mm pitch single-threaded implant in the collar part increased by 50.75% in D2, 65.82% in D3, and 76.79% in D4, whereas that for the 2.4 mm pitch single-threaded implant in the collar region increased by 53.19% in D2, 81.40% in D3, and 93.82% in D4.

Under the conditions of the same thread pattern, the comprehensive relative displacement of the 0.8 mm pitch single-threaded implant in collar part increased by 82.79% in D3 bone and 286.31% in D4 bone as compared with that for the same pitch in D2 bone. The comprehensive relative displacement of a 1.6 mm pitch single-threaded implant in the collar part increased by 101.06% in D3 bone and 353.04% in D4 bone, whereas that for a 2.4 mm pitch single-threaded implant in the collar part increased by 116.46% in D3 bone and 388.78% in D4 bone.

(2) In the Angle Group, the 0.8 mm pitch single-threaded implant had minimum comprehensive relative displacement in the collar part, whereas the triple-threaded implant had the maximum. Compared with the 0.8 mm pitch single-threaded implant, the comprehensive relative displacement of the double-threaded implant in the collar part increased by 19.56% in D2 bone, 28.16% in D3 bone, and 33.55% in D4 bone, whereas that for the triple-threaded implant in the collar part increased by 21.50% in D2 bone, 39.26% in D3 bone, and 45.24% in D4 bone.

Under the conditions of the same thread pattern, the comprehensive relative displacement of the double-threaded implant in the collar part increased by 95.94% in D3 bone and 331.52% in D4 bone, whereas that for the triple-threaded implant increased by 109.52% in D3 bone and 361.78% in D4 bone in the same region of the implant.

(3) In the Compactness Group, compared with the 1.6 mm pitch single-threaded implant, the comprehensive relative displacement of the double-threaded implant in the collar part decreased by 20.69% in D2 bone, 22.71% in D3 bone, and 22.46% in D4 bone. Furthermore, compared with the 2.4 mm pitch single-threaded implant, the comprehensive relative displacement of the triple-threaded implant in the collar part decreased by 20.69% in D2 bone and by 23.23% and 25.07% in both D3 and D4 bone.

### 3.3. Horizontal Load

**Figure 6:**
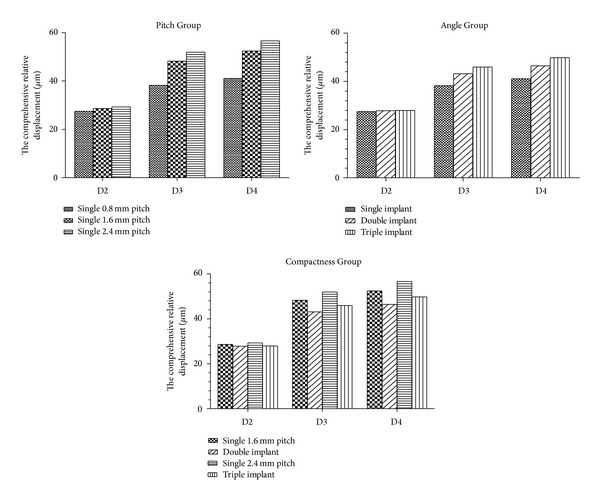
Comprehensive relative displacement among the Pitch Group, the Angle Group, and the Compactness Group under horizontal load.

For the displacement of five implants under horizontal load, please refer to Table 4 and Figure 6. The results of each group are described separately.

(1) In the Pitch Group, the 0.8 mm pitch, single-threaded implant had minimum comprehensive relative displacement in the collar part, whereas the 2.4 mm pitch single-threaded implant had the maximum. With the same pitch, compared with the 0.8 mm pitch single-threaded implant, the comprehensive relative displacement of the 1.6 mm pitch single-threaded implant in the collar part increased by 3.95% in D2 bone, 26.41% in D3 bone, and 27.66% in D4 bone, whereas that for the 2.4 mm pitch single-threaded implant increased by 6.55% in D2 bone, 36.00% in D3 bone, and 37.97% in D4 bone.

Under the conditions of the same thread pattern, the comprehensive relative displacement of 0.8 mm pitch single-threaded implant in the collar part increased by 38.81% in D3 bone and 49.23% in D4 bone as compared with that for the same pitch in D2 bone. The comprehensive relative displacement of the 1.6 mm pitch single-threaded implant in the collar part increased by 68.82% in D3 bone and 83.26% in D4 bone. The comprehensive relative displacement of the 2.4 mm pitch single-threaded implant in the collar part increased by 77.19% in D3 bone and 93.23% in D4 bone.

(2) In the Angle Group, the 0.8 mm pitch single-threaded implant had the minimum comprehensive relative displacement in the collar part, whereas the triple-threaded implant had the maximum. Compared with 0.8 mm pitch single-threaded implant, the comprehensive relative displacement of the double-threaded implant in the collar part increased by 1.19% in D2 bone and by 12.91% and 13.10% in both D3 and D4 bones, whereas that for the triple-threaded implant increased by 1.61% in D2 bone and by 20.29% and 21.31% in both D3 and D4 bones.

Under the conditions of the same thread pattern, the comprehensive relative displacement of the double-threaded implant in the collar part increased by 54.90% in D3 bone and 66.80% in D4 bone. The comprehensive relative displacement of the triple-threaded implant in the collar part increased by 64.34% in D3 bone and 78.15% in D4 bone.

(3) In the Compactness Group, compared with the 1.6 mm pitch single-threaded implant, the comprehensive relative displacements of the double-threaded implant in the collar part decreased by 2.65%, 10.68%, and 11.40% in D2, D3, and D4 bones, respectively. Compared with the 2.4 mm pitch single-threaded implant, the comprehensive relative displacement of the triple-threaded implant in the collar part decreased by 4.63%, 11.55%, and 12.08% in D2, D3, and D4 bones, respectively.

## 4. Discussion

The use of the FEA method in this mechanical analysis of dental implants has been described by many authors [27–29]. In the present study, the 3D FEA method is used to investigate the influence of implant thread design on micromotion level in three different types of bone.

Comprehensive relative displacement of the implant (in* x*-,* y*-, and* z*-axes) was adopted as a parameter to measure micromotion changes. As compared with previous micromotion research, this offers a more accurate method of comparison. To guarantee the comparability of the model, four coordinate points were defined to the collar, tip, and corresponding bone type in each type of implant model, with the points for the collar and the tip the same in all implant models. Four coordinate points were used as nodes in model meshing, each located in the same position in each model. The resonance frequency analysis (RFA) method has also been adopted in previous micromotion research [24, 25], which obtains an RFA value, which is sensitive to changes in bone type and therefore reflects micromotion changes indirectly. Compared with RFA, comprehensive relative displacement directly reflects changes in micromotion of the implant.

In the FEA method, the mesh division is its essence. Therefore, a high-quality mesh division can greatly improve the calculation accuracy. In previous work [27–29], models have been meshed predominantly using FEA software, such as ABAQUS. However, some of the more complicated models do not adopt the FEA software to mesh the elements but adopt the preprocessing software such as Hypermesh 7.0 and then import the data into the FEA software for analysis. The strengths of the FEA software lie in the calculation, solving, and analysis aspects of the modeling, and it is therefore relatively weak in the mesh division aspect of a complicated model. In the present research, the preprocessing software Hypermesh 7.0 was adopted, which builds finite element model through meshing of the line and surface. In this method, the line is first meshed into elements to form two-dimensional surface elements. Then, the 2D surface elements are used to create 3D elements; thus, an increase in element calculation methods generally leads to more accurate calculations. After meshing the elements for the same model with Hypermesh and ABAQUS software, we showed that the elements meshed with Hypermesh software were more likely to be regular, with no deformed elements upon element inspection (data not shown).

In our study, we observed a relative displacement in the vertical direction of the implant under vertical load. In comparison, under horizontal load, the relative displacements occurred in both vertical and horizontal directions. At the same osteotomy site, the vertical displacement was determined to be more than the horizontal displacement. The comprehensive relative displacement at the implant collar under horizontal load was significantly more than that under vertical load, which is in accordance with the clinical presumption of the higher influence of horizontal loading on implant stability [42]. The comprehensive relative displacement of the horizontally loaded implant occurs mainly at the collar, with little movement at the tip (data not shown). Irrespective of the vertical or horizontal load, the comprehensive relative displacement of the implants gradually increased along with the reduction in bone density (D2 > D3 > D4). This finding is in agreement with earlier work [43].

The micromotion of implants with different pitches (the Pitch Group) was compared. Under vertical and horizontal load, the 0.8 mm pitch single-threaded implant had the minimum comprehensive relative displacements at the collar, whereas the 2.4 mm pitch implant had the maximum displacement in all three-bone types. This demonstrates that variation in pitch affects the vertically and horizontally loaded implant in terms of its stability and that as the pitch increases, the implant resistance to vertical and horizontal load diminishes. Our result is consistent with the results of previous studies mentioned above [19].

There are two major differences in implants with different pitches—the helix angle and the compactness—which both affect the stability of the implant. To date, the direct elucidation of the effect of the helix angle and compactness on the micromotion, however, has not been reported. In the present study, we sought to illustrate the effect of the helix angle and compactness on micromotion. Micromotion of identical implants, with a constant pitch of 0.8 mm but different thread helices (Angle Group), was compared. The double- and triple-threaded implants had twice and triple the thread helix of the single-threaded implant, respectively. Irrespective of the load, the 0.8 mm pitch single-threaded implant showed minimum comprehensive relative displacement at the collar, whereas the triple-threaded implant had the maximum. Overall, these results demonstrate that helix angle affects the stability of a vertically and horizontally loaded implant and that, as the thread helix angle increases, the implant resistance to vertical and horizontal load reduces.

The introduction of double- and triple-threaded implants (where two or three threads run parallel to one another [18]) provided implants with theoretically faster insertion times and a reduction in heat generation whilst maintaining a favorable pitch distance for mechanical strength at the bone-implant interface; for example, a triple-threaded implant, with a pitch distance of 0.8 mm, will be inserted 2.4 mm with each 360° rotation. One study identified that these types of implants should be indicated in Type IV cancellous bone [44]. However, it has to be considered that the increase in the number of parallel threads will change the thread helix angle. Sykaras et al. [44] considered that as the helix angle in double- and triple-threaded implants increases, a higher torque is required for placement and thus tighter contact with bone. However, as a viscoelastic material, bone will lose the above-mentioned prestress over time, a process referred to as stress relief [45]. Previous studies have assessed the time and patterns of the prestress release in various viscoelastic materials simulating bone and have shown that 93% is released in 100 hours, that prestress relaxation satisfies a 2-stage function (Kohlrausch-Williams-Watts (KWW) function and the exponential (Debye) function), and that most of the prestress is released in the first 24 hours of this first stage [46–48]. The releasing time in different types of bone is different, as cortical bone is less viscoelastic than cancellous bone [49]. In D4 bone, the release rate of prestress will therefore be faster.

To date, a direct elucidation of stress relief with implant placement in bone has not been reported. However, some authors [50–53] have suggested that ISQ values are statistically lower at the third week after implant placement in all bone types. Barewal and colleagues [50] found that the percentage decrease in stability from baseline to 3 weeks was the highest in D4 bone. At this stage, there are reasons to believe that the faster insertion of implants with double or even triple-threaded implants may actually compromise the final implant success.

In this study, another important pattern emerged for implants with the same helix angle but different thread compactness (Compactness Group). Irrespective of bone quality or load, the comprehensive relative displacement in the collar part of double-threaded implants was smaller than that for 1.6 mm pitch single-threaded implants. Additionally, the comprehensive relative displacement in the collar part of the triple-threaded implants was smaller than that for 2.4 mm pitch single-threaded implants. These findings demonstrate that, with the same threaded helix angle, the resistance of the implant to vertical and horizontal loads enhances as the compactness increases. This is likely to be attributed to the improved mechanic interlocking caused by the increase in the thread compactness, which, in turn, leads to less micromotion and better stability.

**Figure 7:**
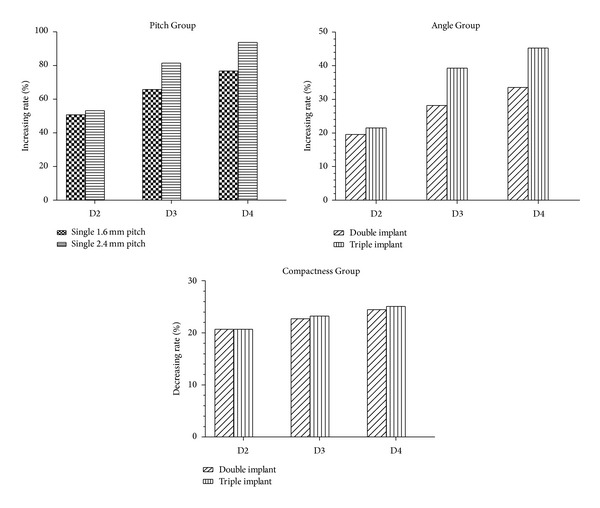
Increasing rates of comprehensive relative displacement between the Pitch Group and the Angle Group under vertical load (comparing the 0.8 mm pitch single-threaded implant). Decreasing rates of comprehensive relative displacement in the Compactness Group (double-threaded implant compared with the 1.6 mm pitch single-threaded implant, and the triple-threaded implant compared with the 2.4 mm-pitch single-threaded implant).

**Figure 8:**
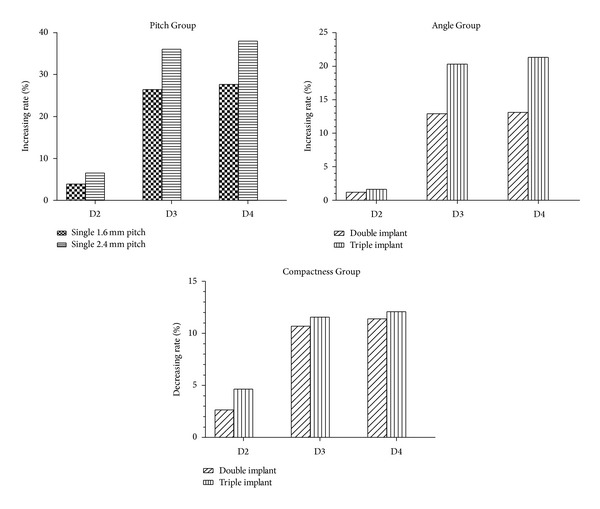
Increasing rates of comprehensive relative displacement between the Pitch Group and the Angle Group under horizontal load (comparing the 0.8 mm pitch single-threaded implant). Decreasing rates of comprehensive relative displacement in the Compactness Group (double-threaded implant compared with the 1.6 mm pitch single-threaded implant, and the triple-threaded implant compared with the 2.4 mm pitch single-threaded implant).

We also noticed that, under vertical load and as compared with the 0.8 mm pitch single-threaded implant, the comprehensive relative displacement of the 1.6 mm pitch and 2.4 mm pitch single-threaded implants in the collar part increased by 50.75% and 53.19% in D2, 65.82% and 81.40% in D3, and 76.79% and 93.82% in D4, respectively (Figure 7). In contrast, under horizontal loading and as compared with 0.8 mm pitch single-threaded implants, the comprehensive relative displacement of 1.6 mm pitch and 2.4 mm pitch single-threaded implants in the collar part increased by 3.95% and 6.55% in D2, 26.41% and 36.00% in D3, and 27.66% and 37.97% in D4, respectively (Figure 8). Compared with the Pitch Group, a similar result in the Angle Group and the Compactness Group was observed. The findings indicate that the influence of thread pitch, helix angle, and compactness on implant stability under vertical load is more effective than under horizontal load. These results suggest that the influence of thread pitch, helix angle, and compactness on implant stability is limited in D2 but that, in D3, which had a reduction in the thickness of the cortical bone, the influence of thread pattern on implant stability was increased under horizontal load. It was originally hypothesized in this study that bone with a thicker cortical component (D2) would be more effective in providing implant stability than thread pattern. However, in D4, with a reduced trabecular bone density as compared with that in D3, there is little increase in implant micromotion under horizontal load between the two bone types. These results provide additional evidence that the density of trabecular bone has less effect on implant micromotion than the thickness of cortical bone under horizontal load. Corresponding with the results obtained in our study, some authors [54–56] also suggest that cortical bone thickness, rather than trabecular bone thickness, plays a more crucial role in implant primary stability.

In our study, the comprehensive relative displacement of the 2.4 mm pitch single-threaded implant in the collar part of the implant in D4 increased by 388.78% and 93.23%, respectively, under vertical and horizontal loads as compared with that of the same implant in D2. However, the comprehensive relative displacement of the 0.8 mm pitch single-threaded implant in the collar part of the implant in D4 increased by 286.31% and 49.23% under vertical and horizontal loads, respectively, compared with that for the same implant in D2. Compared with the Pitch Group, similar results were also found in the Angle Group and the Compactness Group. Overall, our results indicate that the influence of thread pitch, helix angle, and compactness on the comprehensive relative displacement is amplified along with the variation of the bone quality.

In this numerical study, several assumptions were made in the development of the model in the present study. The structures in the models were all assumed to be homogenous and isotropic and to possess linear elasticity. The properties of the materials modeled in this study, particularly the living tissues, however, are different. For instance, it is well documented that the cortical and cancellous bones are neither homogeneous nor isotropic. A frictional contact of bone-implant interface was assumed. Therefore, the results of this study must be interpreted cautiously, and the inherent limitations of 3D FEA studies shall also be considered.

## 5. Conclusion

In conclusion, vertical relative displacement is affected by thread pitch, helix angle, and compactness. Displacement is positively correlated with thread pitch and helix angle but negatively correlated with compactness under vertical loading in variable types of bone density. Under horizontal loading in higher density D2 bone, the thicker cortical component would be more effective in providing implant stability than thread pattern; however, in D3 and D4, with reduced density and increased cancellous structure, the density of trabecular bone has less effect on implant micromotion than the thickness of cortical bone. The influence of thread pitch, helix angle, and compactness on the comprehensive relative displacement is amplified along with the variation of the bone quality under vertical and horizontal loads.

## Figures and Tables

**Table 1:** Experiment grouping.

Group number	Group name	Description	Component
1	Pitch Group	Different pitches	Single 0.8 mm pitch
			Single 1.6 mm pitch
			Single 2.4 mm pitch

2	Angle Group	Same thread compactness but different helix angles	Double-threaded
			Triple-threaded
			Single 0.8 mm pitch

3	Compactness Group	Same helix angle but different thread compactness	Double-threaded and Single 1.6 mm pitch
			Triple-threaded and Single 2.4 mm pitch

**Table 2:** Mechanical properties of the finite element models.

Materials	Young's modulus (*E*, GPa)	Poisson's ratio (*V*)
Compact bone	14.7 [30]	0.3
Dense trabecular bone (for D2, D3 bone)	1.47 [30]	0.3
Low-density trabecular bone (for D4 bone)	0.231 [30]	0.3
Titanium	110 [31, 32]	0.35

**Table 3:** Displacement of five implants under vertical load (*μ*m).

Thread pattern	D2		D3		D4	
	VD	CD	VD	CD	VD	CD
Single-threaded						
Single 0.8 mm pitch	4.797	4.8	8.537	8.774	18.329	18.543
Single 1.6 mm pitch	7.146	7.236	14.401	14.549	32.711	32.782
Single 2.4 mm pitch	7.29	7.353	15.873	15.916	35.785	35.940
Double-threaded	5.676	5.739	11.026	11.245	24.663	24.765
Triple-threaded	5.758	5.832	12.174	12.219	26.773	26.931

VD: the vertical relative displacement; CD: the comprehensive relative displacement; D2–D4: varying types of bone (D2 has the highest density; see Section 2).

**Table 4:** Displacement of five implants under horizontal load (*μ*m).

Thread pattern	D2			D3			D4		
	VD	HD	CD	VD	HD	CD	VD	HD	CD
Single-threaded									
Single 0.8 mm pitch	20.028	18.856	27.512	29.712	25.748	38.192	31.370	26.448	41.056
Single 1.6 mm pitch	21.388	18.984	28.598	37.142	30.053	48.278	39.620	31.405	52.410
Single 2.4 mm pitch	22.284	19.022	29.314	40.736	32.090	51.941	43.119	33.200	56.644
Double-threaded	20.282	18.850	27.84	33.497	26.838	43.124	35.257	28.193	46.436
Triple-threaded	20.490	19.006	27.956	35.833	27.121	45.943	38.035	29.641	49.804

VD: the vertical relative displacement; HD: the horizontal relative displacement; CD: the comprehensive relative displacement; D2–D4: varying density of bone (D2 has the highest density; see Section 2).

## References

[1] Brånemark PI, Hansson BO, Adell R (1977). Osseointegrated implants in the treatment of the edentulous jaw. Experience from a 10-year period. *Scandinavian Journal of Plastic and Reconstructive Surgery: Supplementum*.

[2] Adell R, Lekholm U, Rockler B, Branemark PI (1981). A 15-year study of osseointegrated implants in the treatment of the edentulous jaw. *International Journal of Oral Surgery*.

[3] Albrektsson T, Zarb G, Worthington P, Eriksson AR (1986). The long-term efficacy of currently used dental implants: a review and proposed criteria of success. *The International Journal of Oral & Maxillofacial Implants*.

[4] Wennström JL, Ekestubbe A, Gröndahl K, Karlsson S, Lindhe J (2005). Implant-supported single-tooth restorations: a 5-year prospective study. *Journal of Clinical Periodontology*.

[5] Attard NJ, Zarb GA (2005). Immediate and early implant loading protocols: a literature review of clinical studies. *The Journal of Prosthetic Dentistry*.

[6] Nkenke E, Fenner M (2006). Indications for immediate loading of implants and implant success. *Clinical Oral Implants Research*.

[7] Misch CE, Wang H, Misch CM, Sharawy M, Lemons J, Judy KWM (2004). Rationale for the application of immediate load in implant dentistry: part I. *Implant Dentistry*.

[8] Ioannidou E, Doufexi A (2005). Does loading time affect implant survival? A meta-analysis of 1,266 implants. *Journal of Periodontology*.

[9] Avila G, Galindo P, Rios H, Wang HL (2007). Immediate implant loading: current status from available literature. *Implant Dentistry*.

[10] Tarnow D, Emtiaz SH, Classi A (1997). Immediate loading of threaded implants at stage 1 surgery in edentulous arches: ten consecutive case reports with 1 to 5 year data. *The International Journal of Oral & Maxillofacial Implants*.

[11] Pilliar RM, Lee JM, Maniatopoulos C (1986). Observations on the effect of movement on bone ingrowth into porous-surfaced implants. *Clinical Orthopaedics and Related Research*.

[12] Viceconti M, Muccini R, Bernakiewicz M, Baleani M, Cristofolini L (2000). Large-sliding contact elements accurately predict levels of bone-implant micromotion relevant to osseointegration. *Journal of Biomechanics*.

[13] Szmukler-Moncler S, Salama H, Reingewirtz Y, Dubruille JH (1998). Timing of loading and effect of micromotion on bonedental implant interface: review of experimental literature. *Journal of Biomedical Materials Research*.

[14] Ivanoff C, Gröndahl K, Sennerby L, Bergström C, Lekholm U (1999). Influence of variations in implant diameters: a 3- to 5-year retrospective clinical report. *International Journal of Oral and Maxillofacial Implants*.

[15] Brunski JB (1988). Biomechanical considerations in dental implant design. *The International Journal of Oral Implantology*.

[16] Misch CE (2005). *Dental Implant Prosthetics*.

[17] Geng JP, Ma XX (1995). A differential mathematical model to evaluate side-surface of an Archimede implant. *Shanghai Shengwu Gongcheng Yixue*.

[18] Steigenga JT, al-Shammari KF, Nociti FH, Misch CE, Wang HL (2003). Dental implant design and its relationship to long-term implant success. *Implant Dentistry*.

[19] Akkocaoglu M, Uysal S, Tekdemir I, Akca K, Cehreli MC (2005). Implant design and intraosseous stability of immediately placed implants: a human cadaver study. *Clinical Oral Implants Research*.

[20] O'Sullivan D, Sennerby L, Meredith N (2000). Measurements comparing the initial stability of five designs of dental implants: a human cadaver study. *Clinical implant dentistry and related research*.

[21] Kido H, Schulz EE, Kumar A, Lozada J, Saha S (1997). Implant diameter and bone density: effect on initial stability and pull-out resistance.. *The Journal of oral implantology*.

[22] da Cunha HA, Francischone CE, Filho HN, de Oliveira RCG (2004). A comparison between cutting torque and resonance frequency in the assessment of primary stability and final torque capacity of standard and TiUnite single-tooth implants under immediate loading. *International Journal of Oral and Maxillofacial Implants*.

[23] Kawahara H, Kawahara D, Hayakawa M, Tamai Y, Kuremoto T, Matsuda S (2003). Osseointegration under immediate loading: biomechanical stress-strain and bone formation—resorption. *Implant Dentistry*.

[24] Bischof M, Nedir R, Szmukler-Moncler S, Bernard J, Samson J (2004). Implant stability measurement of delayed and immediately loaded implants during healing. *Clinical Oral Implants Research*.

[25] Meredith N (1998). Assessment of implant stability as a prognostic determinant. *International Journal of Prosthodontics*.

[26] Nedir R, Bischof M, Szmukler-Moncler S, Bernard JP, Samson J (2004). Predicting osseointegration by means of implant primary stability: a resonance-frequency analysis study with delayed and immediately loaded ITI SLA implants. *Clinical Oral Implants Research*.

[27] Chang P, Chen Y, Huang C, Lu W, Tsai H (2012). Distribution of micromotion in implants and alveolar bone with different thread profiles in immediate loading: a finite element study. *The International Journal of Oral & Maxillofacial Implants*.

[28] Fazel A, Aalai S, Rismanchian M, Sadr-Eshkevari P (2009). Micromotion and stress distribution of immediate loaded implants: a finite element analysis. *Clinical Implant Dentistry and Related Research*.

[29] Hsu JT, Fuh LJ, Lin DJ, Shen Y, Huang H (2009). Bone strain and interfacial sliding analyses of platform switching and implant diameter on an immediately loaded implant: experimental and three-dimensional finite element analyses. *Journal of Periodontology*.

[30] Holmes DC, Loftus JT (1997). Influence of bone quality on stress distribution for endosseous implants. J Prosthet Dent. *Journal of Prosthetic Dentistry*.

[31] Lin C-L, Wang J-C, Kuo Y-C (2006). Numerical simulation on the biomechanical interactions of tooth/implant-supported system under various occlusal forces with rigid/non-rigid connections. *Journal of Biomechanics*.

[32] Benzing UR, Gall H, Weber H (1995). Biomechanical aspects of two different implant-prosthetic concepts for edentulous maxillae.. *The International Journal of Oral & Maxillofacial Implants*.

[33] Lekholm U, Zarb GA, Brånemark PI, Zarb GA, Albrektsson T (1985). Patient selection and preparation. *Tissue-Integrated Prostheses: Osseointegration in Clinical Dentistry*.

[34] Linkow LI, Rinaldi AW, Weiss WW, Smith GH (1990). Factors influencing long-term implant success. *The Journal of Prosthetic Dentistry*.

[35] Bass SL, Triplett RG (1991). The effects of preoperative resorption and jaw anatomy on implant success: a report of 303 cases. *Clinical Oral Implants Research*.

[36] Hutton JE, Heath MR, Chai JY (1995). Factors related to success and failure rates at 3-year follow-up in a multicenter study of overdentures supported by Brånemark implants. *The International Journal of Oral & Maxillofacial Implants*.

[37] Mellal A, Wiskott HW, Botsis J, Scherrer SS, Belser UC (2004). Stimulating effect of implant loading on surrounding bone: comparison of three numerical models and validation by *in vivo* data. *Clinical Oral Implants Research*.

[38] Wu JC, Chen C, Yip S, Hsu M (2012). Stress distribution and micromotion analyses of immediately loaded implants of varying lengths in the mandible and fibular bone grafts: a three-dimensional finite element analysis. *The International Journal of Oral & Maxillofacial Implants*.

[39] Ding X, Zhu X-H, Liao S-H, Zhang X-H, Chen H (2009). Implant-bone interface stress distribution in immediately loaded implants of different diameters: a three-dimensional finite element analysis. *Journal of Prosthodontics*.

[40] Mericske-Stern R, Zarb GA (1996). In vivo measurements of some functional aspects with mandibular fixed prostheses supported by implants. *Clinical Oral Implants Research*.

[41] Graf H, Grassl H, Aeberhard HJ (1974). A method for measurement of occlusal forces in three directions. *Helvetica Odontologica Acta*.

[42] Gapski R, Wang H, Mascarenhas P, Lang NP (2003). Critical review of immediate implant loading. *Clinical Oral Implants Research*.

[43] Wang K, Li DH, Guo JF, Liu BL, Shi SQ (2009). Effects of buccal bi-cortical anchorages on primary stability of dental implants: a numerical approach of natural frequency analysis. *Journal of Oral Rehabilitation*.

[44] Sykaras N, Iacopino AM, Marker VA, Triplett RG, Woody RD (2000). Implant materials, designs, and surface topographies: their effect on osseointegration: a literature review. *International Journal of Oral and Maxillofacial Implants*.

[45] Iyo T, Maki Y, Sasaki N, Nakata M (2004). Anisotropic viscoelastic properties of cortical bone. *Journal of Biomechanics*.

[46] Sasaki N, Nakayama Y, Yoshikawa M, Enyo A (1993). Stress relaxation function of bone and bone collagen. *Journal of Biomechanics*.

[47] Goto T, Sasaki N, Hikichi K (1999). Early stage-stress relaxation in compact bone. *Journal of Biomechanics*.

[48] Yetkinler DN, Litsky AS (1998). Viscoelastic behaviour of acrylic bone cements. *Biomaterials*.

[49] Lakes RS, Katz JL (1979). Viscoelastic properties of wet cortical bone—II. Relaxation mechanisms. *Journal of Biomechanics*.

[50] Barewal RM, Oates TW, Meredith N, Cochran DL (2003). Resonance frequency measurement of implant stability in vivo on implants with a sandblasted and acid-etched surface. *International Journal of Oral and Maxillofacial Implants*.

[51] Ersanli S, Karabuda C, Beck F, Leblebicioglu B (2005). Resonance frequency analysis of one-stage dental implant stability during the osseointegration period. *Journal of Periodontology*.

[52] Boronat López A, Balaguer Martínez J, Lamas Pelayo J, Carrillo García C, Peñarrocha Diago M (2008). Resonance frequency analysis of dental implant stability during the healing period. *Medicina Oral Patologia Oral y Cirugia Bucal*.

[53] Sim CPC, Lang NP (2010). Factors influencing resonance frequency analysis assessed by Osstell mentor during implant tissue integration: I. Instrument positioning, bone structure, implant length. *Clinical Oral Implants Research*.

[54] Su Y, Wilmes B, Hönscheid R, Drescher D (2009). Application of a wireless resonance frequency transducer to assess primary stability of orthodontic mini-implants: an in vitro study in pig ilia.. *The International Journal of oral & Maxillofacial Implants*.

[55] Rozé J, Babu S, Saffarzadeh A, Gayet-Delacroix M, Hoornaert A, Layrolle P (2009). Correlating implant stability to bone structure. *Clinical Oral Implants Research*.

[56] Yoon HG, Heo SJ, Koak JY, Kim SK, Lee SY (2011). Effect of bone quality and implant surgical technique on Implant Stability Quotient (ISQ) value. *Journal of Advanced Prosthodontics*.

